# Neuropsychiatric manifestations in inflammatory neuropathies: A systematic review

**DOI:** 10.1002/mus.25112

**Published:** 2016-05-10

**Authors:** Yusuf A. Rajabally, Stefano Seri, Andrea E. Cavanna

**Affiliations:** ^1^ School of Life and Health Sciences, Aston Brain CentreAston UniversityAston TriangleBirminghamB4 7ETUK

**Keywords:** chronic inflammatory demyelinating polyneuropathy, Guillain–Barré syndrome, inflammatory, neuropsychiatric, neuropathy, POEMS syndrome

## Abstract

We conducted a systematic literature review on psychological and behavioral comorbidities in patients with inflammatory neuropathies. In Guillain–Barré syndrome (GBS), psychotic symptoms are reported during early stages in 30% of patients. Typical associations include mechanical ventilation, autonomic dysfunction, inability to communicate, and severe weakness. Anxiety and depression are frequent comorbidities. Anxiety may increase post‐hospital admissions and be a predictor of mechanical ventilation. Posttraumatic stress disorder may affect up to 20% of ventilated patients. Sleep disturbances are common in early‐stage GBS, affecting up to 50% of patients. In chronic inflammatory demyelinating polyradiculoneuropathy, memory and quality of sleep may be impaired. An independent link between depression and pretreatment upper limb disability and ascites was reported in POEMS (polyneuropathy, organomegaly, endocrinopathy, M‐protein, skin) syndrome, with an association with early death. Hematological treatment of POEMS appears effective on depression. Published literature on psychological/behavioral manifestations in inflammatory neuropathies remains scarce, and further research is needed. *Muscle Nerve* **54**: 1–8, 2016

**Abbreviations**
AIDP: acute inflammatory demyelinating polyneuropathy
ASCT: autologous stem cell transplant
BDI: Beck Depression Inventory
CES‐D: Center for Epidemiologic Studies Depression Scale
CIDP: chronic inflammatory demyelinating polyneuropathy
CSF: cerebrospinal fluid
DASS‐21: Depression and Anxiety Stress Scale 21
FIS: Fatigue Impact Scale
FSS: Fatigue Severity Scale
GBS: Guillain–Barré syndrome
GHQ‐28: General Health Questionnaire
HADS: Hospital Anxiety and Depression Scale
MMN: multifocal motor neuropathy
ODSS: Overall Disability Sum Score
ONLS: Overall Neuropathy Limitation Score
POEMS: polyneuropathy, organomegaly, endocrinopathy, M‐protein, skin
IES‐R: Impact of Event Scale—Revised
PCLS: Post‐Traumatic Check List Scale
PHQ‐9: Patient Health Questionnaire
PIPP: Perceived Impact of Problem Profile
PSQI: Pittsburgh Sleep Quality Index
PTSD: posttraumatic stress disorder
REM: rapid‐eye movement
SF‐36: Health‐Related Quality of Life Short Form‐36
STAI‐Y1: State‐Trait Anxiety Inventory Y1
VAS: visual analog score
VEGF: vascular endothelial growth factor

Inflammatory neuropathies include a broad and heterogeneous spectrum of conditions that share in common focal, multifocal, or generalized sensory and/or motor deficits, characterized by acute, progressive, or relapsing and remitting courses.^1^ Treatment strategies depend on the clinical manifestations. Symptomatic therapies are mainly offered to control pain and other sensory symptoms, whereas the aim of immunomodulation is to improve or restore motor and/or sensory function.^1^

Patients with inflammatory neuropathy, irrespective of subtype, can develop neuropsychiatric manifestations that result in significant psychosocial difficulties.^2^ The incidence, characteristics, implications, and consequences of such disturbances are generally not well known, routinely assessed, or adequately considered in routine care of patients with inflammatory neuropathy. Neuropsychiatric symptoms may also add significant burden to health‐related quality of life and have a negative impact on its clinical manifestations, and can include pain and impaired sensory function and motor performance.^3^ Furthermore, it is likely that these aspects may have significant implications for therapeutic efficacy and its objective assessment.

We conducted a systematic review of the scientific literature on the neuropsychiatric presentations in patients with inflammatory neuropathies, namely Guillain–Barré syndrome (GBS), chronic inflammatory demyelinating polyradiculoneuropathy (CIDP), multifocal motor neuropathy (MMN), and paraproteinemic neuropathies, including their different subtypes. We aimed to critically appraise the use of neuropsychiatric/psychological assessment protocols in clinical practice, both in terms of baseline functional evaluations and measurement of therapeutic benefit.

## METHODS

Our search methodology followed the standard guidelines for systematic literature reviews outlined in the PRISMA statement.^4^ We conducted a Medline search of all English language articles published between January 1966 and January 2016, focusing on the psychological and behavioral aspects of all forms of inflammatory neuropathy. We used Medline with the MeSH search terms “inflammatory neuropathy,” “Guillain–Barré syndrome,” “GBS,” “acute inflammatory demyelinating polyneuropathy,” “AIDP,” “acute motor axonal neuropathy,” “AMAN,” “acute motor and sensory axonal neuropathy,” “AMSAN,” “chronic inflammatory demyelinating polyneuropathy,” “chronic inflammatory demyelinating polyradiculoneuropathy,” “CIDP,” “multifocal motor neuropathy,” “MMN,” “paraproteinemic demyelinating neuropathy” (“PDN”), “POEMS” (“polyneuropathy, organomegaly, endocrinopathy, M‐protein, skin”) syndrome, and “CANOMAD” (“chronic ataxic neuropathy with ophthalmoplegia, M‐protein and disialosyl antibodies”) syndrome, each combined with “neuropsychiatric,” “psychiatric,” “psychology,” “psychological assessment,” “depression,” “anxiety,” “sleep disorders,” and “psychosis.” The review specifically focused on ascertainment of psychological state, treatment effects, and disease monitoring. Fatigue was excluded, as we did not consider this an intrinsically neuropsychiatric manifestation, and recent studies have suggested a neuromuscular basis for fatigue in GBS.^5^ We nonetheless included results of fatigue assessment in studies that captured neuropsychiatric features as a primary or secondary focus. Articles were included without regard to disease subtype or course, sample size, analytical approach, monitoring strategy, assessment battery, or therapeutic procedure. The studies that met these initial selection criteria were read in full‐text version and analyzed in detail with special reference to neuropsychiatric presentations (psychological/behavioral/mental health/sleep disorders), outcomes, and conclusions. Reference lists of retrieved articles were searched for any additional relevant publications in the field. The findings are described here using a descriptive approach.

## RESULTS

We identified a total of 20 original articles with data on neuropsychiatric assessments of patients diagnosed with inflammatory neuropathy, and 1 review article published in 2007.^6^ Of these 20 original articles, 2 reported on the same group of patients in the setting of an interventional study. The original articles we reviewed, which are summarized in Table 1, showed considerable heterogeneity in focus. A large number of studies reported the presence of neuropsychiatric disorders during or after the acute presentation of GBS. For clarity, we grouped the studies based on their main focus.

**Table 1:** Studies on neuropsychiatric manifestations in inflammatory neuropathies (Medline search of articles 1966 to January 2016).

Study	Neuropathy subtype(s)	Number of participants	Main findings
Eisendrath *et al*.^7^	GBS	8	Anxiety (100%), hallucinations (75%), depression (87.5%)
Chemtob and Herriott^8^	GBS	1	Posttraumatic stress disorder
Weiss *et al*.^9^	GBS	49	Anxiety (82%), depression (67%), brief reactive psychosis (25%), catatonic psychosis (14%)
Cochen *et al*.^11^	GBS	139	Mental status abnormalities (31%) (visual hallucinations, delusions, dreams)
Garssen *et al*.^12^	GBS	80	Ineffectiveness of amantadine for tiredness
Graham *et al*.^13^	GBS, CIDP	14	Greater anxiety and depression in patients than in controls (significance unknown), improved anxiety (significant) and depression (non‐significant) with exercise
Bussmann *et al*.^14^	GBS, CIDP	20	Anxiety and depression comparable with controls, fatigue severity greater than in controls
Tagami *et al*.^15^	GBS	1	Severe anxiety and depression in fulminant Guillain–Barré syndrome after *Hemophilus influenzae* infection
Khan *et al*.^16^	GBS	76	Higher prevalence of anxiety, depression, and stress than in controls
Bernsen *et al*.^18^	GBS	85	Psychological distress and depressive symptoms present but improved between 3 and 6 months, normalizing at 6 months
Davidson *et al*.^19^	GBS	884	Greater fatigue severity and anxiety in subjects with minor symptoms
Sharshar *et al*.^21^	GBS	110	Anxiety (21%), correlation of anxiety with dyspnea
Witsch *et al*.^22^	GBS	110	Anxiety and depression in 50%, fatigue severity score >5.5 in >30%
Karkare *et al*.^23^	GBS	60	Poor sleep (22%), anxiety and depression (about 40%)
Ranjani *et al*.^25^	GBS	90	Anxiety but not depression correlating with fatigue
Le Guënnec *et al*.^24^	GBS	13	Posttraumatic stress disorder (22%)
dos Santos *et al*.^28^	CIDP	41	Mean MMSE of 26 of 30, memory deficits in 1 of 3, daytime sleepiness in 1 of 3
Zhang *et al*.^26^	POEMS	72	>70% with at least mild depression at baseline, association of depression with upper limb disability and ascites; improvement with treatment of POEMS without antidepressants

GBS, Guillain–Barré syndrome; CIDP, chronic inflammatory demyelinating polyneuropathy, MMSE, Mini‐Mental State Examination; POEMS, polyneuropathy, organomegaly, endocrinopathy, M‐protein, skin.

### Anxiety, Depression, Stress, and Psychotic Symptoms

A prospective study was published in 1983 by Eisendrath *et al*. on 8 GBS patients.^7^ No formal psychological testing was used, and evaluations were subjective and unblinded. All patients reported moderate to severe anxiety and fear intermittently during their stay in the intensive care unit (ICU). Fear of ventilator dysfunction appeared to be common. Anxiety was thought to be improved by staff and family support. Six patients remembered visual hallucinations. These were usually described as frightening and were occasionally accompanied by disorientation. They were common during the plateau phase and were considered to be typical of those reported in ICU patients. Depression was noted in 7 patients during the recovery phase and was thought to be linked to realization of slow recovery and long convalescence.

A case report of posttraumatic stress disorder (PTSD) in a 24‐year‐old woman after severe GBS was published in 1994.^8^ The authors observed that the PTSD in this patient had the same clinical features as when it followed other traumatic events. Symptoms had started more than 3 years after GBS, and the link with the neuropathy was only established after several consultations. The patient was thought to have a specific predisposition due to previous health‐related psychological traumas.

After an initial report of 10 subjects published in German and therefore not included in this review,^9^ a larger prospective study on 49 GBS patients admitted to the ICU was undertaken by Weiss *et al*., who investigated the presence of psychological disturbances in the acute phase of the disease.^10^ Anxiety was present in 82% of patients, depression in 67%, brief reactive psychosis in 25%, and catatonic psychosis in 14%. Psychosis was found to be strongly associated with severe tetraparesis, mechanical ventilation, and multiple cranial nerve involvement. Those having all 3 had an 85% likelihood of experiencing psychotic symptoms. Interestingly, cerebrospinal fluid (CSF) protein levels correlated with the presence of psychotic symptoms. Brain imaging obtained in 7 of 12 psychotic patients did not show abnormalities. When interviewed after their hospital stay, patients described loss of communication as the most stressful problem. When evaluating the subjective experience of ICU stay, 55% of patients felt reassured by the ICU environment, whereas 35% had long‐lasting distress due to their ICU stay. Ninety percent described regular visits from relatives as very helpful to cope with the psychological distress induced by their disease.

In 2005, Cochen *et al*. published a prospective, controlled study of mental status abnormalities and their determinants in a large investigation of 139 French GBS patients and 55 control patients admitted to the ICU with other conditions.^11^ Thirty‐one percent of GBS patients experienced mental status abnormalities that developed at a median time from onset of 9 days, and lasted a median of 8 days, with a maximum duration of 133 days. Of those affected, 60% (corresponding to nearly 20% the whole cohort) had visual hallucinations, mainly described as tiny, colorful, and moving figures. Seventy percent had delusions, mostly of the paranoid type, whereas illusions were present in 30% of GBS patients who were aware of visual, tactile, or auditory manifestations. A further 19% had vivid, emotional, and colorful dreams, with accurate recollection on awakening that often persisted months later. Mental status abnormalities were consistently associated with autonomic dysfunction, disease severity, need for mechanical ventilation, CSF protein levels, and CSF hypocretin‐I levels. Moreover, independent associations with mechanical ventilation and CSF protein levels were identified. These findings led the authors to conclude that the mental status abnormalities experienced by GBS patients are different from ICU delirium and that their most relevant associated features were the presence of autonomic dysfunction, severe GBS, and possibly a transitory hypocretin‐1 transmission decrease.

In a randomized, controlled, crossover trial of amantadine versus placebo for fatigue in GBS,^12^ Garssen *et al*. reported no difference in Fatigue Severity Scale (FSS) changes in severely fatigued GBS patients. With relevance to the current review, Hospital Anxiety and Depression Scale (HADS) scores were used as a secondary outcome measure, and there were no significant changes in the 2 treatment arms. However, individual scores were not provided, and therefore no useful baseline data were available.

In a prospective study of the effects of physiotherapist‐prescribed community‐based exercise on 10 GBS patients at least 1 year post‐diagnosis and 4 patients with stable CIDP, Graham *et al*. used the HADS and FSS as secondary outcome measures.^13^ The primary outcome measure, the Overall Disability Sum Score (ODSS), improved posttreatment by 1 point from a baseline level of 3 (*P* = 0.023) and by a further point at 6 months (*P* = 0.008). The HADS Anxiety score improved from a median of 6 (compared with 4 in normal controls) by 2 points (*P* = 0.02) and by a further 2 points at 6 months (*P* = 0.04). The HADS Depression score improved from a median of 1.5 (compared with 1 in normal controls) by a median of 1 point (*P* = 0.04) and by a further median of 0.5 point at 6 months (*P* = 0.08). It was not specified whether baseline differences in HADS scores were significant between patients and controls. FSS improved from a median of 4.6 points at baseline (significantly higher than in controls, median = 3.4) by a median of 0.6 point post‐exercise (*P* = 0.009) and by a further median of 0.6 point at 6 months (*P* = 0.006).

Bussmann *et al*. reported the results of a study on the effects of physical exercise in 16 patients with previous GBS and 4 patients with stable CIDP.^14^ Both the FSS and HADS were used, as well as the Health‐Related Quality of Life Short Form‐36 (SF‐36) questionnaire and the Rotterdam Handicap Scale. Measurements were obtained at baseline and after a targeted intervention consisting of 3 supervised cycle training sessions on a weekly basis for a total of 12 weeks. Mean HADS scores were within normal range at baseline (mean score = 3.5); however, they improved after the physical exercise intervention (mean score = 3.0). Changes in HADS score showed a strong correlation with changes in muscle power and percentage of active time per 24 hours, as well as the physical component of the Fatigue Impact Scale (FIS). On the other hand, baseline FIS ratings revealed a marked increase in fatigue symptom severity compared with healthy controls [6.1 vs. 2.3 (SD = 0.7)]. The active intervention resulted in a considerable improvement to a mean value of 5.4. Changes in the FIS scores also correlated with changes in percentage of active time.

One case of insomnia, general fatigue, anxiety, and depression in the setting of fulminant GBS after *Hemophilus influenzae* infection was described by Tagami *et al*., with electrically non‐excitable nerves and anti‐GM1 and GD1a antibodies.^15^ Depression persisted despite selective serotonin reuptake inhibitor treatment and required long‐term psychological support.

Khan *et al*. investigated factors affecting long‐term health‐related outcomes in 76 subjects who presented with GBS.^16^ According to the Perceived Impact of Problem Profile (PIPP), both mood and satisfaction with life were substantially affected in 22% of subjects. Anxiety (22.4%), depression (18.4%), and stress (17.1%) were reported with higher prevalence than in a control group of healthy individuals. Specifically, women had significantly higher levels of anxiety, depression, and stress than men, as measured by both Depression and Anxiety Stress Scale 21 (DASS‐21) and PIPP. Unsurprisingly, older patients reported higher PIPP scores on both self‐care and relationships domains, although these ratings did not correlate with anxiety, depression, or stress. Interestingly, time since GBS diagnosis (<6 years or >6 years) had no significant effect on mood. From the point of view of clinical severity indicators, ICU admission, length of stay, MRC scores, or discharge destination did not predict subsequent development of affective symptoms. Based on their findings, the authors concluded that GBS requires long‐term management of psychological sequelae affecting levels of activity and participation.

In a subsequent randomized, controlled trial of high and low‐intensity rehabilitation for late‐stage GBS,^17^ the same group did not find any difference in psychological outcome measures as evaluated by the DASS‐21, although they supported a modest benefit for reduction in motor disability. Interestingly, using the PIPP scale, significant improvement within the relationship domain was observed following intervention (not included in Table 1).

Bernsen *et al*. studied 85 Dutch subjects participating in an international double‐blind RCT comparing intravenous immunoglobulin and placebo, for presence and course of psychological distress, depressive symptoms, and health status at 3, 6, and 12 months after onset.^18^ They used the 28‐item version of the General Health Questionnaire (GHQ‐28) and the Center for Epidemiologic Studies Depression Scale (CES‐D) to assess current mental state and measure of psychological distress and depressive symptoms. Health status was assessed by the Sickness Impact Profile, consisting of physical and psychosocial dimensions. They found that, although psychological distress and depressive symptoms were present and more severe/frequent than in the general population in the early stages, symptoms improved from 3 to 6 months and normalized at 6 months. However, although there was gradual improvement, psychosocial health status was still impaired at 12 months. Anxiety scores remained surprisingly normal throughout the year of study, possibly related to the delayed first assessment at month 3 or use of anxiolytic therapy.

Davidson *et al*.^19^ collected outcome data on general mobility, FSS, HADS, and SF‐36 in a UK postal survey of GBS patients. Of the 1,535 patients contacted, 884 questionnaires (57.6%) were returned. Mean FSS scores were significantly higher in subjects with minor symptoms who were able to run (101 subjects) when compared with healthy controls (median scores of 4.8 vs. 3.2; *P* < 0.001). The group with minor symptoms had higher anxiety levels (median of 6 vs. 4; *P* = 0.012) and depression (median of 4 vs. 1.5; *P* < 0.001) than healthy controls. All domains of the SF‐36, including the mental domain, were significantly more affected in the minor symptom group compared with healthy controls. These patients did not appear to have differences in anxiety and depression ratings in relation to receiving physiotherapy treatment at discharge, as was shown in a separate study by the same authors (not included in Table 1).^20^

Anxiety at ICU admission for GBS was the focus of a study by Sharshar *et al*.^21^ In their prospective, single‐center analysis, 110 patients were assessed for intensity and clinical features of anxiety on admission using the State‐Trait Anxiety Inventory Y1 (STAI‐Y1) and the dyspnea visual analog score (VAS) to investigate whether anxiety was predictive of subsequent respiratory failure. STAI‐Y1 scores were >60/80 in 23 patients (21%), and the dyspnea VAS was >7 of 10 in 28 patients (26%); the 2 measures were also significantly correlated (*P* < 0.0001). Arm disability grade, female gender, disability grade, and presence of bulbar dysfunction correlated with STAI‐Y1 ratings. Moreover, STAI‐Y1 scores were significantly higher in patients who subsequently required mechanical ventilation; these patients considered the uncertainty to be most stressful, whereas patients who did not require mechanical ventilation more often reported pain or weakness as greatest stress‐generators. Interestingly, feelings of uncertainty (rather than severity of anxiety) were most strongly associated with respiratory failure.

The long‐term (defined as ≥12 months) outcome of GBS patients who required mechanical ventilation was further investigated by Witsch *et al*.^22^ Approximately 65% of survivors had pain at the time of interview, and nearly 50% had anxiety and depression. Over 30% had significant fatigue, with an FSS score of >5.5. Neither anxiety/depression nor fatigue were significantly associated with age. There was an unexpected association with treatment type; all these neuropsychiatric symptoms had better outcomes after intravenous immunoglobulin as compared with plasma exchange, but the reasons are unclear. Administration of 1 versus multiple immunoglobulin courses had no further impact on subsequent psychological state and fatigue severity.

Karkare *et al*. studied anxiety and depression as secondary outcome measures, using the HADS, in their sleep study of 60 GBS patients in the acute phase.^23^ They showed that 23 of 60 patients (38.3%) had anxiety, and 24 of 60 (40%) had depression. Further details were not provided.

Le Guënnec *et al*. investigated the impact of prolonged mechanical ventilation by measuring the prevalence of PTSD or posttraumatic stress symptoms (PTSS) in GBS patients.^24^ The Horowitz Impact of Event Scale (IES), the Impact of Event Scale—Revised (IES‐R), and the Post‐Traumatic Check List Scale (PCLS) were used to assess PTSD symptoms. Depression was assessed using the HADS and Beck Depression Inventory (BDI). Only 13 of 22 patients who had been ventilated for at least 2 months could be included in the study; the mean time from weaning from mechanical ventilation to the evaluation was 3 years, with a range between 2 and 5 years. Twenty‐two percent of patients fulfilled the criteria for PTSD according to the *Diagnostic and Statistical Manual of Mental Disorders, 4th ed*. (DSM‐IV). Of note, patients from this small cohort did not report other anxiety or affective symptoms, as measured by psychometric instruments, with median HADS Anxiety subscale score of 5 (range 4–11.5), median HADS Depression subscale score of 1 (range 0–3.5), and median BDI score of 1 (0–5).

Ranjani *et al*. assessed anxiety and depression as correlates of fatigue in GBS in the neurorehabilitation setting using the HADS.^25^ Their study involved 90 subjects and showed that, at discharge, fatigue correlated significantly with anxiety (*P =* 0.042), but not with depression. The study did not provide detailed data on findings relating to anxiety and depression.

Zhang *et al*. recently published a large and comprehensive study on the prevalence and determinants of depression in patients newly diagnosed with POEMS syndrome, a relatively rare multisystem hematological condition that causes a mixed demyelinating and axonal inflammatory neuropathy.^26^ In this study, 72 patients were assessed at baseline using the Patient Health Questionnaire (PHQ‐9) scale and the Overall Neuropathy Limitation Score (ONLS). Patients were subsequently re‐evaluated at 3‐month intervals after different therapeutic interventions, including autologous stem cell transplant (ASCT) and melphalan and dexamethasone treatment. The PHQ‐9 is a 9‐item self‐administered psychometric instrument that assesses depressive symptoms. PHQ‐9 total scores range between 0 and 27, with higher scores indicating more severe depression. Using a cut‐off score of 10, the authors of this study estimated the prevalence of clinical depression at 38.0% (pretreatment). Over 70% of patients reported at least mild depressive symptoms with a PHQ‐9 score >4; however, none were taking antidepressants. Compared to patients without depression, patients with clinical depression had higher ONLS upper limb scores (*P* = 0.03) and lower hemoglobin levels (*P* = 0.04), as well as higher rates of physical conditions, including hypothyroidism (*P* = 0.01), ascites (*P* = 0.01), and pleural effusion (*P* = 0.03). Interestingly, there was no association with serum vascular endothelial growth factor (VEGF) level, a useful disease marker for POEMS. VEGF is a cytokine that has been proposed recently as a marker of severe depression, raising the possibility of a link between immunological changes and development of affective symptoms in the context of POEMS syndrome.^27^ Multivariate logistic regression identified only upper limb ONLS scores (*P* = 0.02) and the presence of ascites (*P* = 0.04) as independent predictors of clinical depression. The authors commented that the association between depression and upper (but not lower) limb ONLS could be due to higher impact of arm disability on psychological well‐being. Pretreatment clinical depression was otherwise shown to be significantly associated with early death (*P =* 0.04). Although no antidepressants were administered, median PHQ‐9 scores decreased significantly after hematological treatment of the underlying disease (*P =* 0.001). Furthermore, incidence of depression dropped from 38% pretreatment to <2% posttreatment. This led the authors to conclude that antidepressant therapy may not be necessary in POEMS patients with pretreatment depression. Interestingly, although steroids may cause depression as a well‐known adverse effect, these agents did not appear to have this effect in patients with POEMS syndrome who received them as part of their treatment.

### Sleep Disorders

Cochen *et al*. studied both quantitative and qualitative sleep parameters, which they found to be poor in all GBS patients; specifically, sleep patterns were fragmented and unstable when patients reported mental status abnormalities.^11^ In addition, GBS patients and mental status abnormalities had major rapid‐eye movement (REM) sleep abnormalities, including shortened REM sleep latencies, REM sleep without atonia, and abnormal bursts of eye movements during non‐REM sleep. They concluded that the sleep abnormalities in GBS suggested that mental status abnormalities can be difficult to differentiate from wakeful dreams caused by underlying sleep disorders.

Karkare *et al*. prospectively studied sleep quality in the acute phase of GBS in 60 patients using a wide range of assessment tools, including the Pittsburgh Sleep Quality Index (PSQI), Richards Campbell Sleep Score, and St. Mary's Hospital Sleep Questionnaire.^22^ Over half of the patients had poor sleep during their hospital stay. They reported that 22% had “poor quality of sleep at baseline” (presumably corresponding to the initial hospital assessment rather than to their pre‐disease status). Sleep disturbance (defined by a Richards Campbell Sleep Score of >33) was significantly associated with anxiety (*P* = 0.009), but not with depression, despite a statistical trend that failed to reach significance (*P* = 0.07).

In a study of CIDP patients by dos Santos *et al*.,^28^ daytime sleepiness was reported by one‐third of the recruited sample, and 1 in 6 reported sleeping only 2–4 hours a night, suggesting a possible impact of their condition on sleep. The main limitation of their study is that the disruption of sleep patterns could only been inferred from self‐reported data, as polysomnography was not available for objective sleep assessment.

Ranjani *et al*. also used the PSQI and reported a 73% rate of sleep disturbance at admission, possibly due to a high prevalence of neuropathic pain (77%) and paresthesia (60%). There was, however, no association with fatigue.^25^

### Cognitive Dysfunction

We found only 1 study of higher cognitive functions in inflammatory neuropathy; it used the Mini‐Mental State Examination (MMSE).^28^ Forty‐one patients with CIDP were studied, and the mean MMSE score was 26 (range 22–30). Although cognitive screening did not suggest significant impairment, one‐third of patients reported memory deficits.

## DISCUSSION

In this study we have reviewed the evidence on neuropsychiatric comorbidities in patients with inflammatory neuropathies. The first relevant finding was the relative paucity of published literature on this topic, which precluded the possibility of complementing this descriptive review with secondary data analysis. Furthermore, the variability in assessment methods, including psychometric scales, absence of normative values, heterogeneity of study focus, and lack of longitudinal data, all represent major limitations in drawing strong conclusions from the available evidence.

Most of the available data come from studies conducted on GBS patients. However, as shown in this review, the variability in timing between the acute manifestation and psychometric assessment is a strong limitation. Available data from the studies by Weiss *et al*.,^10^ Sharshar *et al*.,^21^ and Karkare *et al*.^23^ suggest that anxiety is not an uncommon finding in the context of acute GBS, with a prevalence ranging from 20% at admission to 40%–80% during ICU stay. Specifically, early manifestations of anxiety appear to be associated with bulbar dysfunction and may interestingly predict the need for subsequent mechanical ventilation. Anxiety comorbidity in the longer term is reported with higher frequency when compared with the normal population, affecting over 20% of patients with previous GBS, as shown by Khan *et al*.^16^ Similarly, depression and stress appear to be more frequent in patients with a history of GBS, even years after the acute episode.^15^, ^20^ There is some uncertainty about the duration of psychological distress in view of findings by Bernsen *et al*.^18^ Both anxiety and depression may correlate with the presence of neurological disability and persistence of minor symptoms affecting patient well‐being, even in the presence of complete recovery from GBS,^18^ although there has been no consistency of findings in this regard.^17^ Fatigue severity, as well as the mental component of the SF‐36 and pain symptoms, appeared to be closely associated with degree of functional recovery. Although exclusively a disease of the peripheral nervous system in its classical form, GBS appears to be accompanied by early changes in mental status, including hallucinations and delusions, in as many as one‐third of patients, especially in association with severe weakness, mechanical ventilation, and autonomic dysfunction.^6^, ^7^ This may have a central mechanism, as suggested by the correlation with CSF protein levels found in 2 studies.^9^, ^10^ Loss of voluntary movement and ability to communicate may be contributors to the development of psychotic symptoms.

However, previously reported high rates of “ICU psychosis” or “hyperactive delirium in ICU,” reaching up to 60%–80% in ventilated subjects, could indirectly support its observation in GBS patients.^29^ Also, 30% of ventilated ICU patients have been documented to have long‐term cognitive difficulties up to 6 years after hospital discharge.^30^ This is in keeping with findings in GBS samples.^10^, ^13^ The main differential diagnosis is therefore with ICU psychosis. The evidence in the Cochen *et al*. study shows that mental status abnormalities were twice as frequent in GBS than in ICU controls, despite GBS patients being younger and less exposed to psychoactive drugs and metabolic disorders.^11^ The onset of neuropsychiatric symptoms occurred before ICU admission in 16% of GBS patients. There were no reported risk factors for ICU delirium in these subjects (i.e., aging, metabolic disorders, use of psychoactive drugs, and placement in a windowless ICU area/bay). In addition, the GBS patients had qualitatively more elaborate dreams, illusions, and hallucinations, with more severe and more frequent delusions. All these elements support that GBS‐psychosis and ICU‐psychosis are qualitatively distinct manifestations. However, there is a lack of confirmatory studies, since that of Cochen *et al*., does not allow definite identification of a GBS‐specific psychosis.

In addition, irrespective of ICU admission, the acuteness and severity of the neuropathy in the case of GBS may explain the neuropsychiatric features described. Comparable manifestations have been reported with other disorders. After stroke, which causes similarly acute, severe disability, rates of depression (29%–33%) and anxiety (24%) are higher than in the general population,^31^ and not dissimilar to those reported in several studies in GBS.^16^, ^21^, ^22^, ^23^ Of interest, however, only low percentages of stroke patients, ranging from 0.4% to 3.1%, have a psychotic disorder, with single psychotic symptoms occurring in up to 10% and hallucinations in only 4%.^31^ These figures contrast with the much higher rates reported by Cochen *et al*. in GBS, which supports their hypothesis of a GBS‐specific psychosis.^11^

In practice, appropriate consideration and exclusion of GBS or neuropathy mimics, such as porphyria^32^ and lead intoxication,^33^ remain essential in the presence of neuropsychiatric manifestations, which should not be assumed to necessarily be GBS‐related. Adequate repeated assessments, maintaining communication, and offering targeted treatment for anxiety, depression, and psychosis, should be considered. Sleep disturbances are also common in the acute phase of GBS, possibly affecting up to 50% of patients.^22^ Small case series have suggested both sleep quality and quantity are probably affected, with sleep fragmentation as well as REM sleep abnormalities.^10^ However, studies that focused on sleep problems have also been conducted on very small patient samples, thus limiting the generalizability of the conclusions.^22^

A study on a large cohort of patients with POEMS syndrome, a rare nosological entity, showed high prevalence of pretreatment depression, with figures approaching 40%.^26^ Independent association between depression and upper limb disability and ascites was shown to be a risk factor for early death. Interestingly, treatment of POEMS syndrome without use of antidepressants was highly effective in resolving depression in the vast majority of patients. Unfortunately, no studies have provided data in patients with more common inflammatory neuropathies such as CIDP, except for a single analysis assessing basic cognitive function.^28^ In this review could not identify any relevant data on patients with other inflammatory neuropathies.

In conclusion, neuropsychiatric aspects can be of major importance in patients with inflammatory neuropathies; their occurrence has the potential to influence the severity of the clinical presentation, its functional impact, and health‐related quality of life.^3^ This contrasts with the paucity of studies in this area. As shown in studies on GBS and POEMS syndrome, there are significant associations between neuropsychiatric comorbidities and physical ability, and, importantly for clinical practice, even subtle physical deficits appear related to anxiety and depression. It seems likely that similar features may be present to varying degrees in all types of inflammatory neuropathies. The therapeutic implications of the POEMS syndrome study are of great interest; in addition to the effects on the neuropathy, treatment had additional measurable effects on patients' mood. In CIDP and multifocal motor neuropathy, knowledge of neuropsychiatric symptoms at baseline would be of great interest. Their progression with treatment, together with their actual impact on treatment effectiveness and ease of monitoring, also deserve investigation. These aspects are likely to be of relevance to improving understanding of disease profiles and for implementing more effective management strategies.

Further studies are needed to understand the prevalence and implications of the neuropsychiatric aspects of inflammatory neuropathies. This additional knowledge may improve future global care and management of patients, taking into account this generally poorly understood and neglected aspect of this group of disorders.
